# Combining dense elements with attention mechanisms for 3D radiotherapy dose prediction on head and neck cancers

**DOI:** 10.1002/acm2.13655

**Published:** 2022-06-03

**Authors:** Samuel Cros, Hugo Bouttier, Phuc Felix Nguyen‐Tan, Eugene Vorontsov, Samuel Kadoury

**Affiliations:** ^1^ MedICAL Laboratory Polytechnique Montreal Montréal Canada; ^2^ Centre Hospitalier de l'Université de Montréal (CHUM) Montréal Canada; ^3^ Centre de recherche du CHUM (CRCHUM) Montréal Canada

**Keywords:** attention mechanisms, convolutional neural networks, dense architectures, knowledge‐based planning, radiation dose prediction methods

## Abstract

**Purpose:**

External radiation therapy planning is a highly complex and tedious process as it involves treating large target volumes, prescribing several levels of doses, as well as avoiding irradiating critical structures such as organs at risk close to the tumor target. This requires highly trained dosimetrists and physicists to generate a personalized plan and adapt it as treatment evolves, thus affecting the overall tumor control and patient outcomes. Our aim is to achieve accurate dose predictions for head and neck (H&N) cancer patients on a challenging in‐house dataset that reflects realistic variability and to further compare and validate the method on a public dataset.

**Methods:**

We propose a three‐dimensional (3D) deep neural network that combines a hierarchically dense architecture with an attention U‐net (HDA U‐net). We investigate a domain knowledge objective, incorporating a weighted mean squared error (MSE) with a dose‐volume histogram (DVH) loss function. The proposed HDA U‐net using the MSE‐DVH loss function is compared with two state‐of‐the‐art U‐net variants on two radiotherapy datasets of H&N cases. These include reference dose plans, computed tomography (CT) information, organs at risk (OARs), and planning target volume (PTV) delineations. All models were evaluated using coverage, homogeneity, and conformity metrics as well as mean dose error and DVH curves.

**Results:**

Overall, the proposed architecture outperformed the comparative state‐of‐the‐art methods, reaching 0.95 (0.98) on D95 coverage, 1.06 (1.07) on the maximum dose value, 0.10 (0.08) on homogeneity, 0.53 (0.79) on conformity index, and attaining the lowest mean dose error on PTVs of 1.7% (1.4%) for the in‐house (public) dataset. The improvements are statistically significant (p<0.05) for the homogeneity and maximum dose value compared with the closest baseline. All models offer a near real‐time prediction, measured between 0.43 and 0.88 s per volume.

**Conclusion:**

The proposed method achieved similar performance on both realistic in‐house data and public data compared to the attention U‐net with a DVH loss, and outperformed other methods such as HD U‐net and HDA U‐net with standard MSE losses. The use of the DVH objective for training showed consistent improvements to the baselines on most metrics, supporting its added benefit in H&N cancer cases. The quick prediction time of the proposed method allows for real‐time applications, providing physicians a method to generate an objective end goal for the dosimetrist to use as reference for planning. This could considerably reduce the number of iterations between the two expert physicians thus reducing the overall treatment planning time.

## 1 INTRODUCTION

Head and neck (H&N) cancers develop in regions with several critical structures demonstrating a high degree of appearance variability. The H&N region is a very challenging site due to the proximity of organs at risk of cancers, the presence of intersecting structures, and the need for variable dose levels. Successive technology leaps in external radiation therapy have improved the manner in which this sensitive region is treated. First, intensity‐modulated radiation therapy^[^ ^1^, ^2^, ^3^, ^4^ ^]^ (IMRT) uses varying beam intensities to better reach tumors in intricate areas. Subsequently, volumetric modulated arc therapy^[^ ^5^, ^6^, ^7^, ^8^, ^9^ ^]^ (VMAT) dynamically administers the dose in a spiral pattern around the patient, allowing for a shorter treatment time. Both methods rely on imaging modalities (CT, MRI) for personalized planning that largely improves dose delivery and tumor targeting. However, the rising complexity of the delivery method leads to a significant increase in time to generate dosimetric plans. The standard process of knowledge‐based planning (KBP) involves a time‐consuming process between an oncologist and a dosimetrist that requires iterating between clinically desirable and physically deliverable dose plans. This iterative work requires tremendous expertise and can take several hours to complete.

In recent years, the use of machine learning in radiation oncology has grown exponentially with the rise of deep learning and its numerous applications in computer vision through convolutional neural networks (CNN).^[^ ^10^, ^11^, ^12^, ^13^, ^14^ ^]^ In 2015, Ronneberger et al. proposed the U‐net^[^ ^15^ ^]^ architecture for medical image segmentation. This neural network model is an encoder–decoder architecture with long skip connections from the downsampling encoder to the upsampling decoder that enable a recovery of spatial detail during the upsampling process. First designed for two‐dimensional (2D) image slices, three‐dimensional (3D) variants were developed in the following years,^[^ ^16^, ^17^ ^]^ motivated by the increased availability of volumetric medical imaging data. These 3D implementations proved highly relevant in dose prediction tasks,^[^ ^10^, ^11^ ^]^ as they allow correct dose prediction across 2D slices avoiding notable errors near the boundaries of a planning target volume. Several alternatives have allowed to improve the U‐net training capabilities with the development of residual networks^[^ ^18^, ^19^ ^]^ and dense CNNs,^[^ ^20^, ^21^ ^]^ both aiming for efficient deep networks that would achieve higher performance. However, working with the 3D variant of U‐net requires more memory and computational time.

In 2018, Oktay et al. and Schempler et al. proposed a modular implementation of attention gates (AG) in a standard U‐net architecture (attention U‐net) developed for medical image segmentation tasks.^[^ ^22^, ^23^ ^]^ They relied on additive gates that only filter information flowing through the long skip connections, resulting in a memory‐efficient attention module for the U‐net. This led to improved results and demonstrated the benefit of AG to identify and localize specific structures. In 2019, Nguyen et al. developed a 3D neural network architecture^[^ ^11^ ^]^ that incorporated dense elements within the quintessential U‐net architecture (HD U‐net) while retaining a reasonable memory usage. They demonstrated the superiority of their network compared with their counterparts, namely the standard U‐net and the DenseNet. However, this approach is patch based, which is inherently prone to reconstruction artifacts.

The tendency of the mean‐squared error (MSE) loss for quick convergence regardless of the target domain made it a prime candidate for most deep learning regression problems. However, its inability to capture domain‐specific knowledge remains a strong limitation for medical imaging tasks, especially when dealing with dose prediction which still relies heavily on knowledge‐based and physics‐driven approaches.^[^ ^24^ ^]^ In 2018, Mahmood et al. developed a knowledge‐based dose planning pipeline using adversarial learning.^[^ ^12^, ^13^ ^]^ Even though such an architecture effectively incorporates domain knowledge, it tends to rely on a high number of hyperparameters, making it difficult to train. In 2019, Nguyen et al. proposed a dosimetric objective based on the dose‐volume histogram,^[^ ^14^ ^]^ a commonly used metric and a defining tool in KBP for cancer treatment. They adapted the DVH metric into a differentiable objective and included an adversarial objective to capture the remaining domain knowledge. They observed substantial improvements in dose estimation for prostate cancer, mainly in the form of a reduced trade‐off between planning target volumes (PTV) coverage and sparing of planning target volume (OARs). However, the training time was 2.5 times slower than a typical MSE loss.

We propose a predictive model that produces high‐quality dose plans on annotated volumetric data by introducing a hierarchically dense attention U‐net (HDA U‐net) that can process annotated CT scans with OAR and PTV segmentations. We assess the compatibility between dense‐ and attention‐based elements within a fully connected encoder–decoder‐like architecture to generate dose distributions. We also investigate the integration of a weighted MSE‐DVH loss and validate the method on two separate datasets of H&N cancer patients achieving top performance on each. We compare our network against two state‐of‐the‐art models, namely attention U‐net and HD U‐net, and we assess dose coverage, homogeneity, and conformity performance.

## 2 METHODS AND MATERIALS

### 2.1 Data collection and formatting

Two separate and independent datasets were used to train and validate the proposed architecture: an in‐house clinical dataset and a public dataset used for an open challenge. First, the in‐house dataset consists of 150 H&N patients treated with IMRT or VMAT. The selection criteria for the in‐house dataset included two main categories of plans: curative plans for pharynx and neck cancers and curative plans for advanced oropharynx cancers; it also excluded three categories of dose plans, namely: unilateral doses, highly focused doses, and supplementary doses. These exceptional case scenarios require very specific tuning such as the addition of phantom delineations by the dosimetrist and fell outside the scope of our study. These two categories of plans were adequately represented in both training and testing sets for each seed for the different models. No significant difference was observed in terms of performance between the two categories. The original axial plane resolution was reduced from 512 × 512 to 128 × 128 (1 mm${}^{3}$ voxel size) due to memory constraints during training. This was done via downscaling by a factor of 4 using b‐spline interpolation. Dose plans in full resolution could only be processed in a patch‐based approach, which leads to reconstruction issues at test time. When presented with a patch without a tumor, models tend to predict plans with low levels of gradients and homogeneous dose values, without considering the actual proximity with cancerous tissue just beyond the patch. This is especially relevant in H&N cancers where preventive radiation is commonly sent to regions of future cancer growth. The same problem can occur when using 2D data and has been handled with 2.5D methods that include additional slices as context for the model.^[^ ^25^ ^]^ In our case, downscaling allowed us to use the full 3D data and ensured that the model is presented with broader in‐plane context information as well as an additional cross‐plane axis to build stronger correlations. The final processed images had a resolution of 128 × 128 × [32‐96]. The manually segmented structures include the medullary canal, outer medullary canal, esophagus, oral cavity, mandible, trachea, trunk, outer trunk, left and right parotids, left and right inner ear, left and right eyes, left and right submaxillary glands, left optic nerve as well as the PTV, clinical target volume (CTV), and gross tumor volume (GTV). The percentages of cases for each OAR is as follows: medullary canal (89%), outer medullary canal (80%), esophagus (80%), oral cavity (80%), mandible (81%), trachea (78%), trunk (76%), outer trunk (71%), left parotid (81%), right parotid (80%), left ear (82%), right ear (82%), left eye (75%), right eye (77%), left submax. (70%), right submax. (69%), PTV (87%), CTV (93%), and GTV (70%). Segmentations were performed by experienced radiation oncologists. Patients were prescribed a dose varying from 66 to 70 Gy, which was administered in multiple fractions.

The second dataset is a public dataset available through the OpenKBP challenge.^[^ ^26^ ^]^ We utilized the available 340 H&N patients as 128 × 128 × 128 volumes treated with IMRT only. The manually segmented structures include the brain stem, spinal cord, right and left parotids, esophagus, larynx, mandible as well as the PTV 70, PTV 63, and PTV 56. Segmentations were also performed by experienced radiation oncologists. The latter refers to the high‐dose target volume receiving 70 Gy, the mid‐dose volume receiving 63 Gy, and the low‐dose volume receiving 56 Gy. Each patient was prescribed a dose of 70 Gy administered in 35 fractions.

For both datasets, we included the baseline CT as the first input channel and kept the structure masks in a binary format, where the 1‐values indicate the voxels assigned to the structure and where each structure was represented by a separate channel. In total, the input data were represented by 21 channels for the in‐house dataset (one CT and 20 structure channels) and by 11 channels for the public dataset (one CT and 10 structure channels).

### 2.2 HDA U‐net architecture

#### 2.2.1 Standard U‐net architecture

Standard U‐net architectures^[^ ^15^ ^]^ consist of an encoder that skips features to a decoder at each resolution in the network. The encoder acts as a downsampling path where the original input resolution is successively halved while the number of features grows. Using this strategy, the model progressively integrates more spatial information as its receptive field grows. At each stage in the decoder, the features go through an upsampling operation followed by a convolution and are then combined with features drawn from the encoder. These long skip connections are key in helping the network predict high‐resolution detail.

#### 2.2.2 Dense elements

**FIGURE 1:**
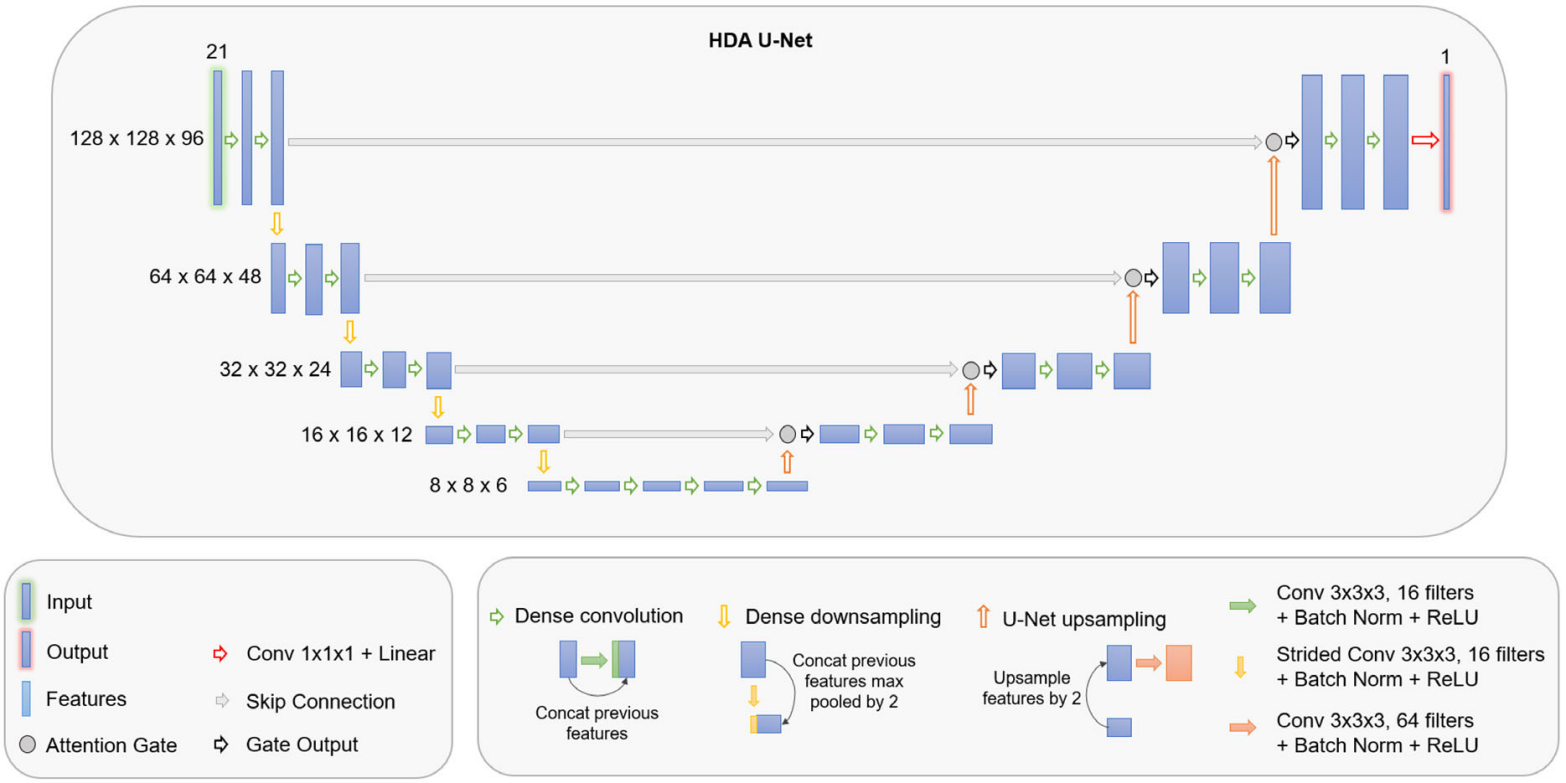
Schematic illustration of the proposed HDA U‐net architecture that combines dense elements with attention mechanisms

The proposed model is based on hierarchically dense connected U‐nets, which are used to capture the different sections of the model within a hierarchy,^[^ ^11^ ^]^ where the input's resolution remains constant—namely between two maximum pooling and two upsampling operations. This type of model introduces two key operations: the dense convolution and the dense downsampling operations. The dense convolution consists of the application of a 3 × 3 × 3 convolution followed by a Rectified Linear Unit (ReLU), the result of which is concatenated to the previous features similarly to short skip connections in a DenseNet architecture.^[^ ^20^ ^]^ This sequence happens twice at each level of the hierarchy except in the bottom‐most stage where it is repeated four times. This sequence of operations is common practice in encoder–decoder architectures as it improves the representation capabilities of the network at a low computational cost given the low input resolution in these layers (8 × 8 × 6 in our case). The dense downsampling operation concatenates the result of a 3 × 3 × 3 convolution with stride 2 followed by a ReLU with the previous features downsampled by a factor of 2 by max pooling. Together, this ensures that the number of features grows linearly with each operation while staying relatively low compared with a standard 3D U‐net architecture.^[^ ^16^ ^]^ The global architecture is presented in Figure 1.

#### 2.2.3 Attention mechanisms

**FIGURE 2:**
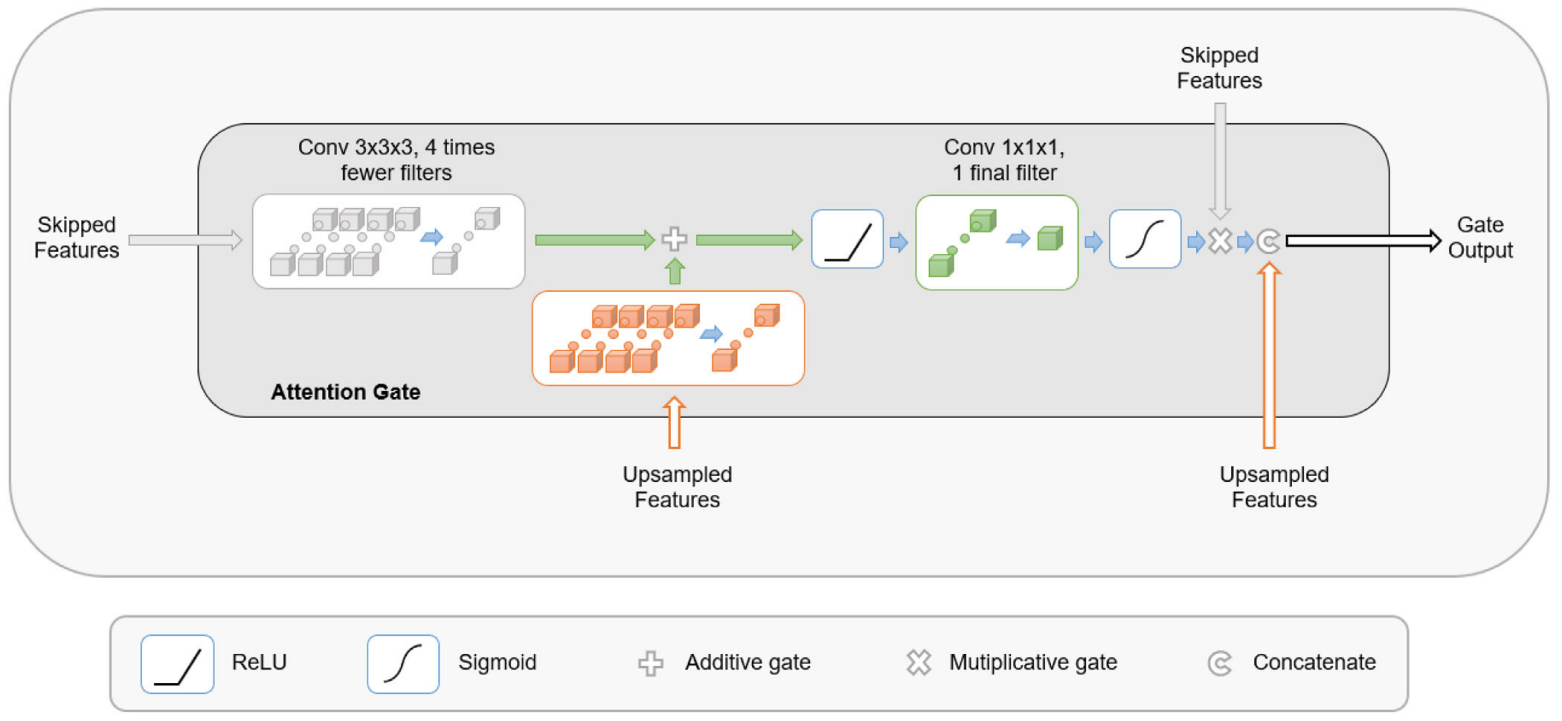
Schematic illustration of the implemented attention gate using skipped features and upsampled features to produce attention coefficients

Soft attention gates specifically designed for U‐nets were incorporated into the proposed architecture.^[^ ^22^, ^23^ ^]^ Figure 2 depicts the implementation of the attention gates used in the proposed model. Instead of directly concatenating the skipped information with the upsampled features, the attention gate aims at tuning coefficients to suppress less relevant activations in the skipped features. More precisely, skipped features and upsampled features are convolved and summed before a ReLU, then the ReLU output is reweighted into attention coefficients in [0, 1]; the exact implementation details can be found in Figure 2. Once the expanding path of the U‐net resumes, the skipped features are multiplied by these coefficients and concatenated with the upsampled features.

#### 2.2.4 Proposed HDA U‐net architecture details

The proposed HDA U‐net architecture implements the dense convolutions and dense downsampling operations, with the addition of a batch normalization layer after each convolution, to produce a combined output, which is then used by the attention gates. The proposed model uses a growth rate of 16 to match the number of features added at each convolution within a hierarchy in the HD U‐net. The number of features entering the attention gate after upsampling and convolution was set to 64 in concordance with previous studies.^[^ ^11^ ^]^

### 2.3 Training and validation

We repeated the experiments randomly initialized using three different random seeds on our in‐house dataset, each time splitting the data into training (70%), validation (15%), and test (15%) subsets. For training on the public data, we used the same training, validation, and test sets as defined in the challenge setup which consists of a training set of 200 patients, a validation set of 40 patients, and a test set of 100 patients. In both cases, the best version of the model's weights was determined by the epoch that obtained the lowest validation loss and then evaluated on the testing data. Both datasets had their input CT images, and output radiation doses were standardized by min‐max normalization.

We implement the differential approximation of the DVH,^[^ ^14^ ^]^ which given a structure s, its associated binary mask $M_{s}$, and a volumetric dose D can be defined as

(1) $$\begin{array}{c} {\tilde{\mathrm{DVH}}}_{s}\left(D,M_{s}\right)=\left(v_{s,d_{1}},v_{s,d_{2}},\text{…},v_{s,d_{n}}\right), \end{array}$$

where the volume $v_{s,d_{t}}$ is a volume v with voxel intensities at or above a threshold value $d_{t}$ for a given structure s, which can be approximated as

(2) $$\begin{array}{c} v_{s,d_{t}}\left(D,M_{s}\right)=\frac{\sum_{i,j,k}\text{Sigmoid}\left(\frac{m}{\beta_{t}}\left(D_{i,j,k}\right)-d_{t}\right))M_{s}\left(i,j,k\right)}{\sum_{i,j,k}M_{s}\left(i,j,k\right)}, \end{array}$$

where $\text{Sigmoid}\left(x\right)=\frac{1}{1+e^{-x}}$ is the sigmoid function, m is a steepness parameter, $\beta_{t}$ is the bin width of the histogram, and i,j,k are voxel indices for the 3D arrays. The t is an index for the dose threshold values and bin widths.

We have chosen a maximum dose threshold $d_{t}$ by searching the range [0, 80] Gy, with an increment step size of 1 Gy. Following the results of previous studies when searching for optimized dose levels, we set the steepness value to m=1. This small value ensures model convergence while retaining a fair approximation of the DVH. More information on parameter choices can be found in the source material.^[^ ^14^ ^]^

This leads to the definition of the DVH objective as

(3) $$\begin{array}{ccc} & & L_{\mathrm{DVH}}\left(D_{\mathrm{true}},D_{\mathrm{pred}},M\right)=\frac{1}{n_{s}}\frac{1}{n_{t}}\\ & & \;\sum_{s}{\left\|{\tilde{\mathrm{DVH}}}_{s}\left(D_{\mathrm{true}},M_{s}\right)-{\tilde{\mathrm{DVH}}}_{s}\left(D_{\mathrm{pred}},M_{s}\right)\right\|}_{2}^{2}, \end{array}$$

where $D_{\mathrm{true}}$ and $D_{\mathrm{pred}}$ are the ground truth and predicted doses, respectively, $n_{s}$ is the number of structures, and $n_{t}$ is the number of threshold values.

This objective was included in the loss function of the networks in a weighted manner as $L_{\mathrm{Total}}=w_{1}L_{\mathrm{MSE}}+w_{2}L_{\mathrm{DVH}}$ with $w_{1}=1$ and $w_{2}=0.1$. These were chosen so that a trained model would exhibit the same magnitude for both terms. Also, we observed that when dealing with additional losses, keeping a strong driving MSE was key in achieving proper convergence. We used the Adam optimizer^[^ ^27^ ^]^ with a learning rate of 1 × $10^{-3}$, a batch size of 1, and default parameters $\beta_{1}$ = 0.9, $\beta_{2}$ = 0.999, ε = 1 × $10^{-7}$. All models were trained for 200 epochs. Neural networks using the simple MSE objective were trained and evaluated using an NVIDIA V100 SXM2 GPU with 16 GB of dedicated RAM. Neural networks using the weighted MSE‐DVH objective were trained and evaluated using an NVIDIA Titan RTX GPU with 24 GB of dedicated RAM.

Table 1 displays information on the total number of parameters, the average training time per epoch, and the average prediction time for each architecture. While the attention U‐net shows around 26M total parameters, both the HD and HDA variants of the U‐net have a considerably lower parameter count of 3.4M and 3.5M, respectively. The dense elements integrated into these models allow for steady growth of 16 feature channels, while the attention U‐net, much like the standard U‐net, doubles the number of features at each level. The average training time is quite similar between the two HD variants at around 142 s per epoch for the in‐house dataset; around 406 s per epoch for the public one. It is however 20–30% higher than the attention U‐net, which demonstrates the true cost of the HD variant in which the higher connectivity leads to longer time spent at updating weights. The same is true for the average prediction times that span between 0.43 and 0.53 s for the in‐house dataset; between 0.67 and 0.88 s on the public dataset. It should be noted that the prediction times reported here do not take into account the machine‐dependent environment loading time (which was around 5–10 s). We decided to present performance measures in a real‐life situation where the environment is already loaded and the machine is ready to receive the patient's CT and segmentations.

**TABLE 1:** Parameters, training, and prediction time for each model

		Average training time per epoch		Average prediction time (excluding environment loading time)	
	Total parameters	In‐house ± 5 s	Public ± 10 s	In‐house ± 0.05 s	Public ± 0.07 s
HD U‐net	3.4M	134 s	402 s	0.50 s	0.74 s
Attention U‐net	26M	120 s	315 s	0.43 s	0.67 s
HDA U‐net	3.5M	141 s	402 s	0.51 s	0.75 s
HD U‐net (DVH)	3.4M	145 s	404 s	0.52 s	0.87 s
Attention U‐net (DVH)	26M	123 s	320 s	0.44 s	0.67 s
HDA U‐net (DVH)	3.4M	148 s	414 s	0.53 s	0.88 s

Abbreviations: DVH, dose‐volume histogram; HD, hierarchically dense; HDA, hierarchically dense attention.

### 2.4 Dosimetric measurements

Models were evaluated using the following dosimetric measurements: coverage values (D99, D98, D95), maximum dose value (Dmax), homogeneity $(H1=\frac{D2-D98}{D50}$ and $H2=\frac{D95}{D50})$, conformity index, van't Riet conformation number^[^ ^28^ ^]^ $\left(\frac{{\left(V_{\mathrm{PTV}}\;\cap \;V_{100\%\mathrm{Iso}}\right)}^{2}}{V_{\mathrm{PTV}}\times V_{100\%\mathrm{Iso}}}\right)$, mean dose error (Dmean), as well as sample DVH curves. The coverage metrics are computed within the tumor, measuring the percentage of predicted dose relative to the prescribed dose, considering the first (D99), second (D98), and fifth (D95) percentile of predicted doses. These metrics ensure that the vast majority of cancerous tissues are treated at a high percentage of the prescribed dose. Similarly, the maximum dose value shows the percentage of the maximum predicted dose relative to the maximum prescribed dose. The homogeneity indices are computed within the PTV as well. They depend on the difference between the worst predicted dose value and the best one, normalized over a median predicted dose value. They convey how homogeneously the tumor is treated across its entire volume. The conformity index and conformation number measure how many voxels meet the expected radiation threshold on the target volumes, while the mean dose error acts as a broad measurement of quality and gives an idea of the error to expect for each structure. Finally, given a dose in Gray, the DVH shows the volume of tissue receiving at least that amount of radiation. It allows to easily verify the satisfaction of constraints on the maximum and minimum dosage for each structure. To measure the statistical significance of these metrics, we performed a two‐tailed student's *t*‐test in Microsoft Excel between the predictions' scores of the proposed method and the predictions' scores of each other presented method.

## 3 RESULTS

The experiments were divided into two categories. The first set of experiments focused on the in‐house dataset using clinical data from our institution, while the second set of experiments was performed on the public data from the OpenKBP challenge.

### 3.1 Clinical in‐house dataset

Table 2 shows the PTV coverage and maximum dose values for the in‐house dataset. All values are reported as a percentage of the prescribed dose. The attention U‐net, the HD U‐net, and the proposed HDA U‐net all receive a noticeable performance increase with the addition of the DVH loss on all coverage metrics. Overall the attention U‐net and the proposed method outperform every other architecture on all coverage metrics. The proposed method does so while maintaining a significantly lower maximum dose value.

**TABLE 2:** Coverage and maximum dose values for each model with ground truth values from the in‐house dataset

Architectures	D99	D98	D95	Dmax
HD U‐net	0.86* ± 0.03	0.88* ± 0.03	0.90* ± 0.02	1.08 ± 0.02
Attention U‐net	0.86* ± 0.03	0.88* ± 0.03	0.90* ± 0.03	1.09* ± 0.03
HDA U‐net	0.89* ± 0.03	0.90* ± 0.02	0.93* ± 0.02	1.07 ± 0.04
HD U‐net (DVH)	0.89* ± 0.02	0.91* ± 0.02	0.93* ± 0.01	1.08* ± 0.01
Attention U‐net (DVH)	0.91 ± 0.01	0.92 ± 0.01	0.95 ± 0.01	1.19* ± 0.18
HDA U‐net (DVH)	**0.92** ± **0.01**	**0.93** ± **0.01**	**0.95** ± **0.01**	**1.06** ± **0.02**
Ground truth	0.96 ± 0.00	0.97 ± 0.00	0.98 ± 0.00	1.03 ± 0.00

*Note*: * shows that values are significantly different than the proposed HDA U‐Net (DVH) results on the test data (*p* < 0.05). The best values among the architectures are shown in bold in each column.

Abbreviations: DVH, dose‐volume histogram; HD, hierarchically dense; HDA, hierarchically dense architecture.

Table 3 shows the homogeneity and conformity values for the in‐house dataset. Similarly, the addition of the DVH loss improves the performance of every architecture. The proposed method significantly outperforms all methods on the homogeneity metrics H1 and H2 and competes with the attention U‐net on the conformity metrics. The performance remains quite far from reference scores for both the conformity index and the van't Riet conformation number regardless of the network.

**TABLE 3:** Homogeneity and conformity values for each model with ground truth values from the in‐house dataset

Architectures	H1	H2	CI	van't Riet
HD U‐net	0.18* ± 0.03	0.87* ± 0.01	0.40* ± 0.06	0.28* ± 0.03
Attention U‐net	0.19* ± 0.01	0.86* ± 0.01	0.44 ± 0.14	0.32* ± 0.13
HDA U‐net	0.15* ± 0.04	0.89* ± 0.03	0.45 ± 0.18	0.33* ± 0.10
HD U‐net (DVH)	0.14* ± 0.02	0.90* ± 0.02	0.41* ± 0.03	0.36* ± 0.03
Attention U‐net (DVH)	0.17* ± 0.09	0.90* ± 0.04	0.50 ± 0.01	0.42 ± 0.06
HDA U‐net (DVH)	**0.10** ± **0.02**	**0.93** ± **0.01**	**0.53** ± **0.05**	**0.45** ± **0.03**
Ground truth	0.06 ± 0.00	0.96 ± 0.00	0.85 ± 0.03	0.70 ± 0.03

*Note*: * shows that values are significantly different than the proposed HDA U‐net (DVH) results on the test data (*p*‐values < 0.05). The best values among the architectures are shown in bold in each column.

Abbreviations: DVH, dose‐volume histogram; HD, hierarchically dense; HDA, hierarchically dense architecture.

**FIGURE 3:**
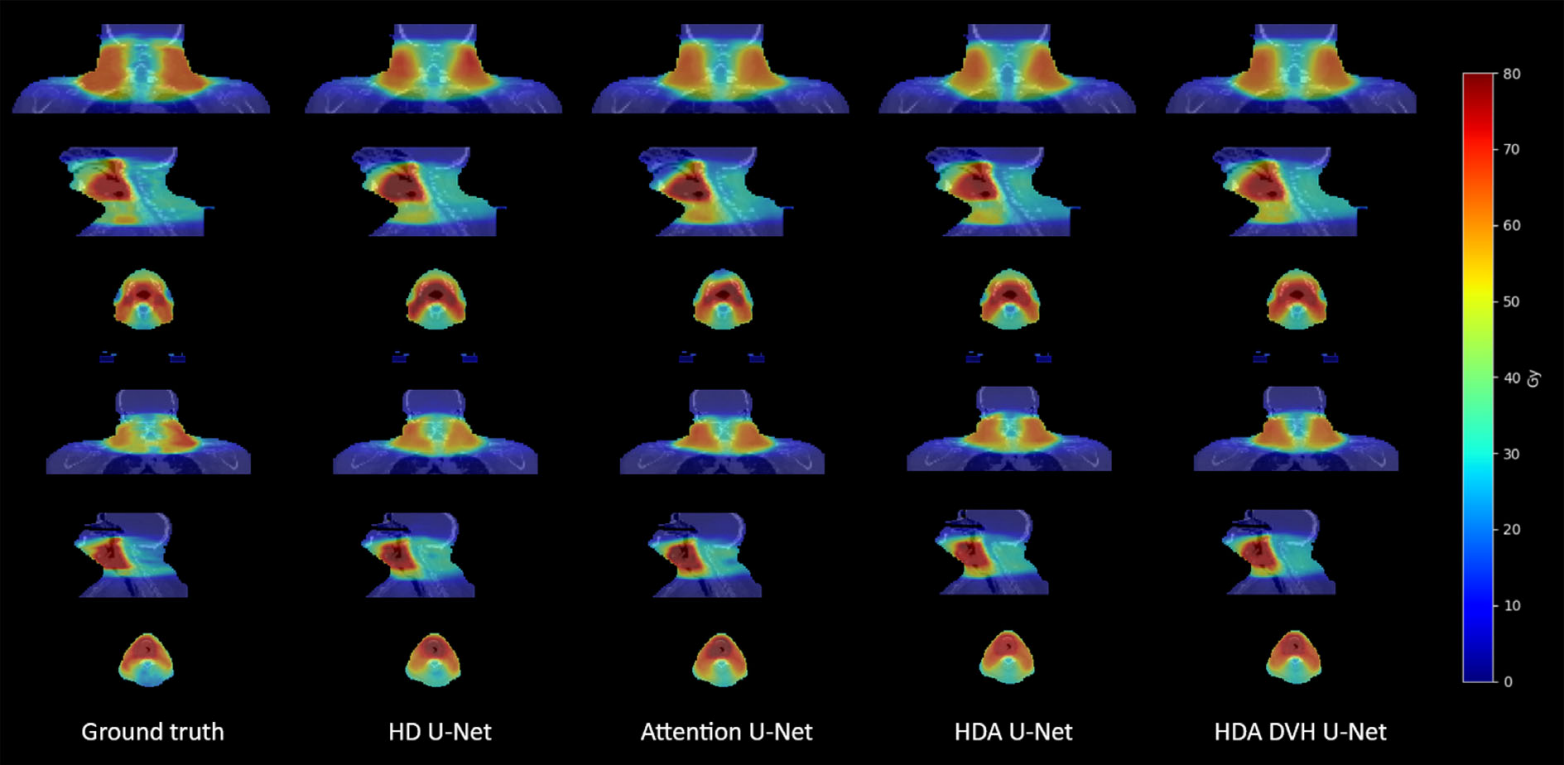
Dose examples overlapped with the input CT for two sample in‐house test patients from ground truth, HD U‐net, attention U‐net, HDA U‐net, and proposed HDA DVH U‐net

Figure 3 shows predicted dose volumes on two sample patients from the in‐house dataset. Every architecture is able to generate visually satisfying plans compared with the reference which justifies the extensive use of several metrics to properly differentiate them. However for the first example patient, we can observe a shortage of dose in Gy around the mouth for the attention U‐net and the HD U‐net, this is more visible on the sagittal and axial views. Overall, the proposed architecture yields the lowest average dose error in both examples with a plan within 3.8% and 2.4% average error of the reference distribution.

**FIGURE 4:**
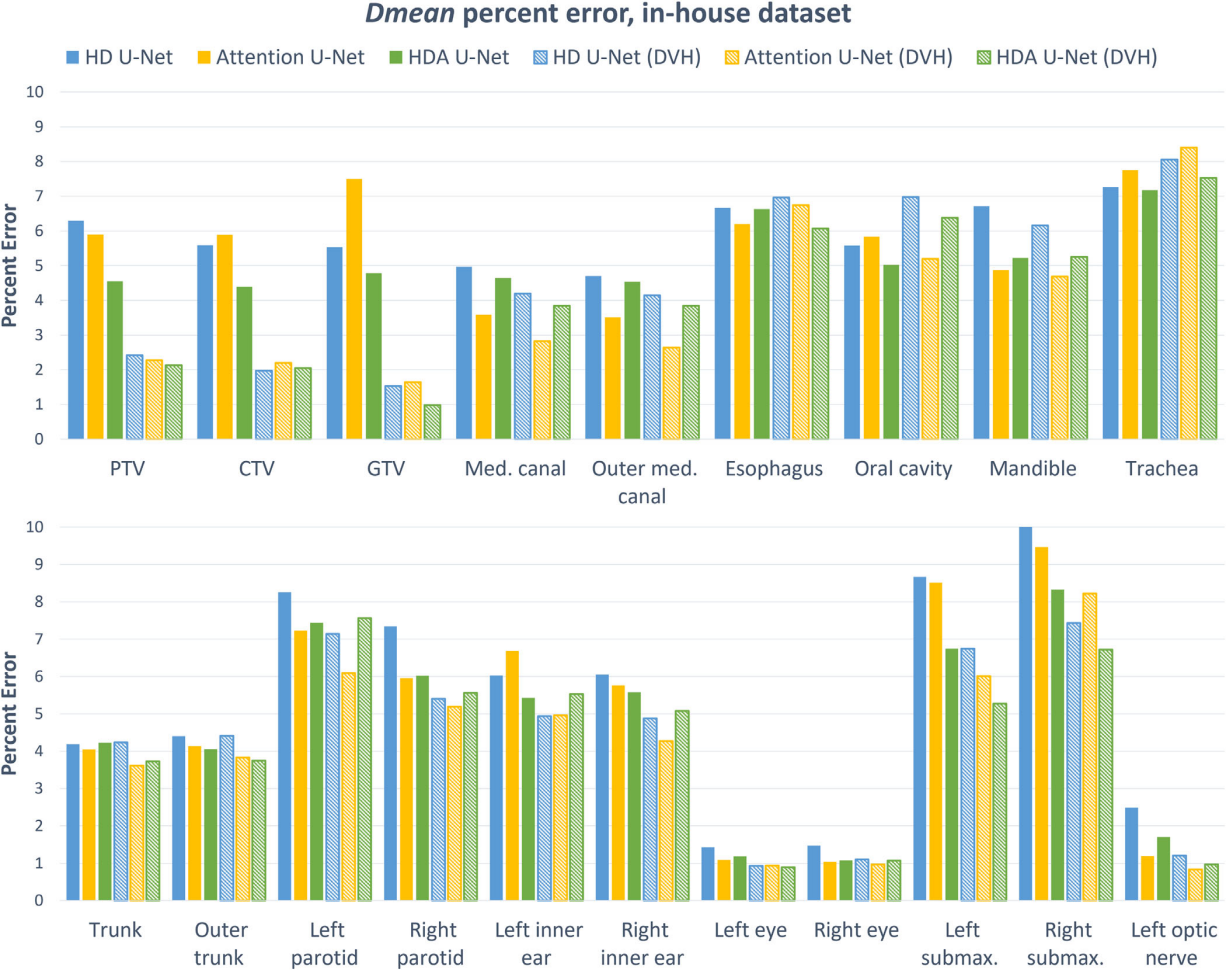
Dose mean percent error per structure for each model on the in‐house dataset

Figure 4 shows the average mean dose error for each architecture, on each structure of the in‐house dataset. These are reported as a percentage of the prescription dose which gives an approximate idea of the quality to expect per structure. The addition of the DVH loss in all three architectures helped reduce the dose error in most structures. We observed up to a 2% reduction in dose error for the submaxillary glands and up to a 4% reduction for the PTV, CTV, and GTV. The attention U‐net yields the lowest error on average on the OARs with a mean dose error of 4.4% (4.6% for the proposed) while the proposed method yields the lowest error on average on TVs with a mean dose error of 1.7% (2.0% for attention U‐net).

**FIGURE 5:**
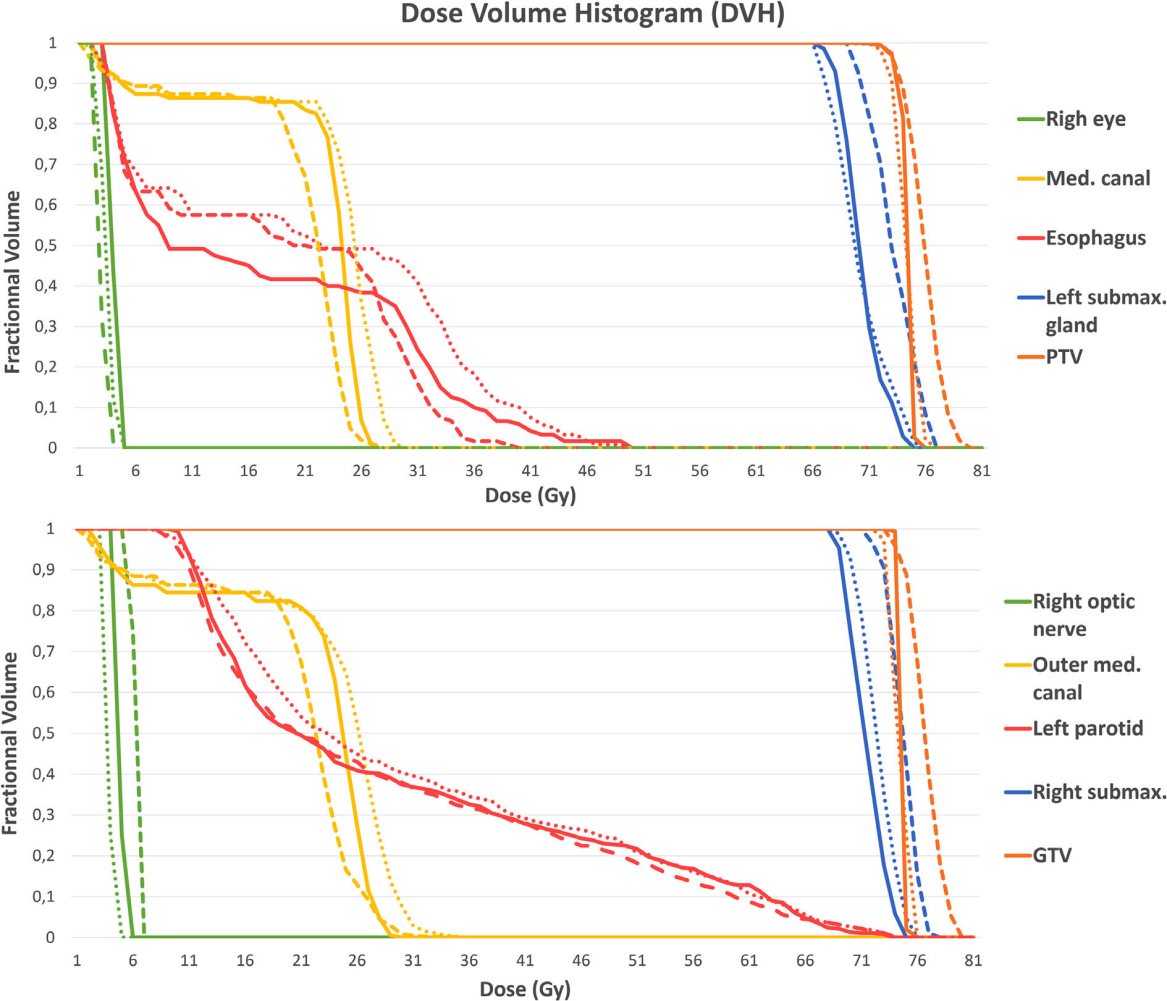
DVH curves for a sample test patient from the in‐house dataset with doses from ground truth (solid line), HDA U‐net (dashed line), and proposed HDA U‐net using DVH loss (dotted line)

Figure 5 shows sample DVH curves from a patient from the in‐house dataset. To demonstrate the influence of the DVH loss on the performance, and for the sake of readability, we have chosen to include only the ground truth (in solid line), the HDA U‐net trained with MSE (in dashed line), and the proposed HDA U‐net trained with a weighted sum of MSE and DVH loss (in dotted line). For this sample patient, the curves of the proposed model are closer to the ground truth ones on a vast majority of structures (with the exception of the esophagus and left parotid).

**FIGURE 6:**
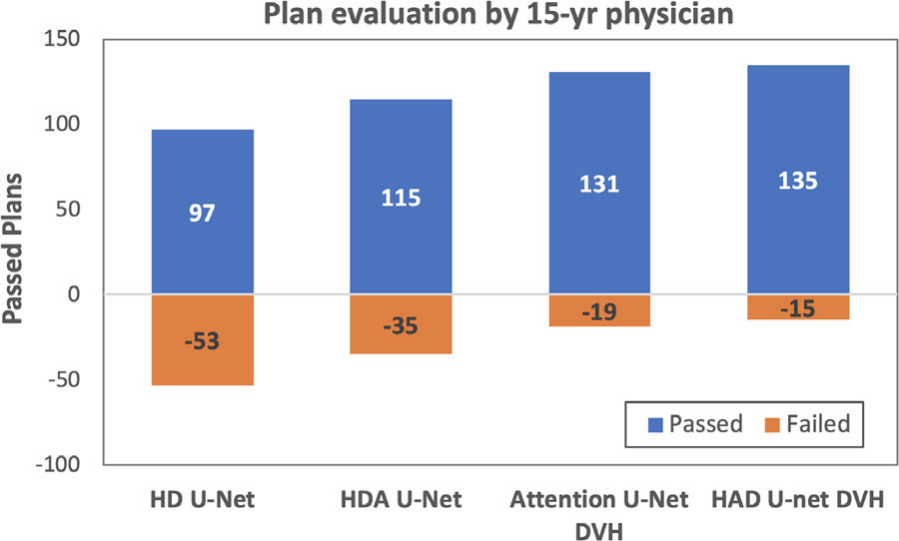
Evaluation of automatic plans by a 15‐year‐experienced physician. (Passed: clinically acceptable. Failed: is not be clinically approved based on PTV dose coverage or inadequate organ sparing)

The 150 dose plans produced by four different deep learning‐based methods (including the HDA U‐net with DVH loss) were assessed by a physician with 15 years of experience in dose planning. Results show that 135/150 plans passed the clinical criteria for the proposed method, which is an improvement compared to the standard HD U‐net, which have 97/150 plans accepted. The results of this evaluation are presented in Figure 6.

### 3.2 OpenKBP challenge public dataset

Table 4 shows the PTV coverage and maximum dose values for the public dataset. The attention U‐net and the proposed HDA U‐net receive a noticeable performance boost with the addition of the DVH loss on all coverage metrics. Overall, the attention U‐net and the HDA U‐net using the DVH loss achieve significantly higher coverage scores than the HD variant. The maximum dose value is similar for all methods except attention U‐net, which scores lower without the DVH loss and higher with it.

**TABLE 4:** Coverage and max dose values for each model with ground truth values from the OpenKBP dataset

Architectures	D99	D98	D95	Dmax
HD U‐net	0.93* ± 0.02	0.94* ± 0.02	0.96* ± 0.01	1.07 ± 0.03
Attention U‐net	0.93* ± 0.03	0.94* ± 0.02	0.96* ± 0.02	1.05* ± **0.02**
HDA U‐net	0.94* ± 0.03	0.95* ± 0.02	0.97* ± 0.02	1.06 ± 0.02
HD U‐net (DVH)	0.92* ± 0.03	0.94* ± 0.03	0.96* ± 0.02	1.07 ± 0.02
Attention U‐net (DVH)	0.94 ± 0.02	0.96 ± 0.02	0.97 ± 0.01	1.10* ± 0.05
HDA U‐net (DVH)	**0.95** ± **0.03**	**0.96** ± **0.02**	**0.98** ± **0.02**	1.07 ± 0.03
Ground truth	0.96 ± 0.05	0.97 ± 0.04	0.99 ± 0.03	1.06 ± 0.03

*Note*: * shows that values are significantly different than the proposed HDA U‐net (DVH) results on the test data (p‐values < 0.05). The best values among the architectures are shown in bold in each column.

Abbreviations: DVH, dose‐volume histogram; HD, hierarchically dense; HDA, hierarchically dense architecture.

Table 5 shows the homogeneity and conformity values for the public dataset. The HD U‐net and the proposed HDA U‐net receive a noticeable performance boost with the addition of the DVH loss on all homogeneity and conformity metrics. The proposed method scores the highest in terms of homogeneity with near ground truth level of quality while retaining reasonable conformity values.

**TABLE 5:** Homogeneity and conformity values for each model with ground truth values from the OpenKBP dataset

Architectures	H1	H2	CI	van't Riet
HD U‐net	0.12* ± 0.03	0.91* ± 0.02	0.67* ± 0.10	0.65* ± 0.10
Attention U‐net	0.09 ± 0.02	0.94 ± 0.01	0.62* ± 0.17	0.60* ± 0.17
HDA U‐net	0.09* ± 0.04	0.93* ± 0.01	0.78 ± 0.12	0.76 ± 0.12
HD U‐net (DVH)	0.10* ± 0.03	0.93* ± 0.02	0.77 ± 0.12	0.76 ± 0.12
Attention U‐net (DVH)	0.09* ± 0.02	0.93* ± 0.01	**0.80** ± **0.10**	**0.78** ± **0.10**
HDA U‐net (DVH)	**0.08** ± **0.02**	**0.94** ± **0.01**	0.79 ± 0.15	0.75 ± 0.14
Ground truth	0.08 ± 0.04	0.96 ± 0.05	0.87 ± 0.19	0.77 ± 0.19

*Note*: * shows that values are significantly different than the proposed HDA U‐Net (DVH) results on the test data (p‐values < 0.05). The best values among the architectures are shown in bold in each column.

Abbreviations: DVH, dose‐volume histogram; HD, hierarchically dense; HDA, hierarchically dense architecture.

**FIGURE 7:**
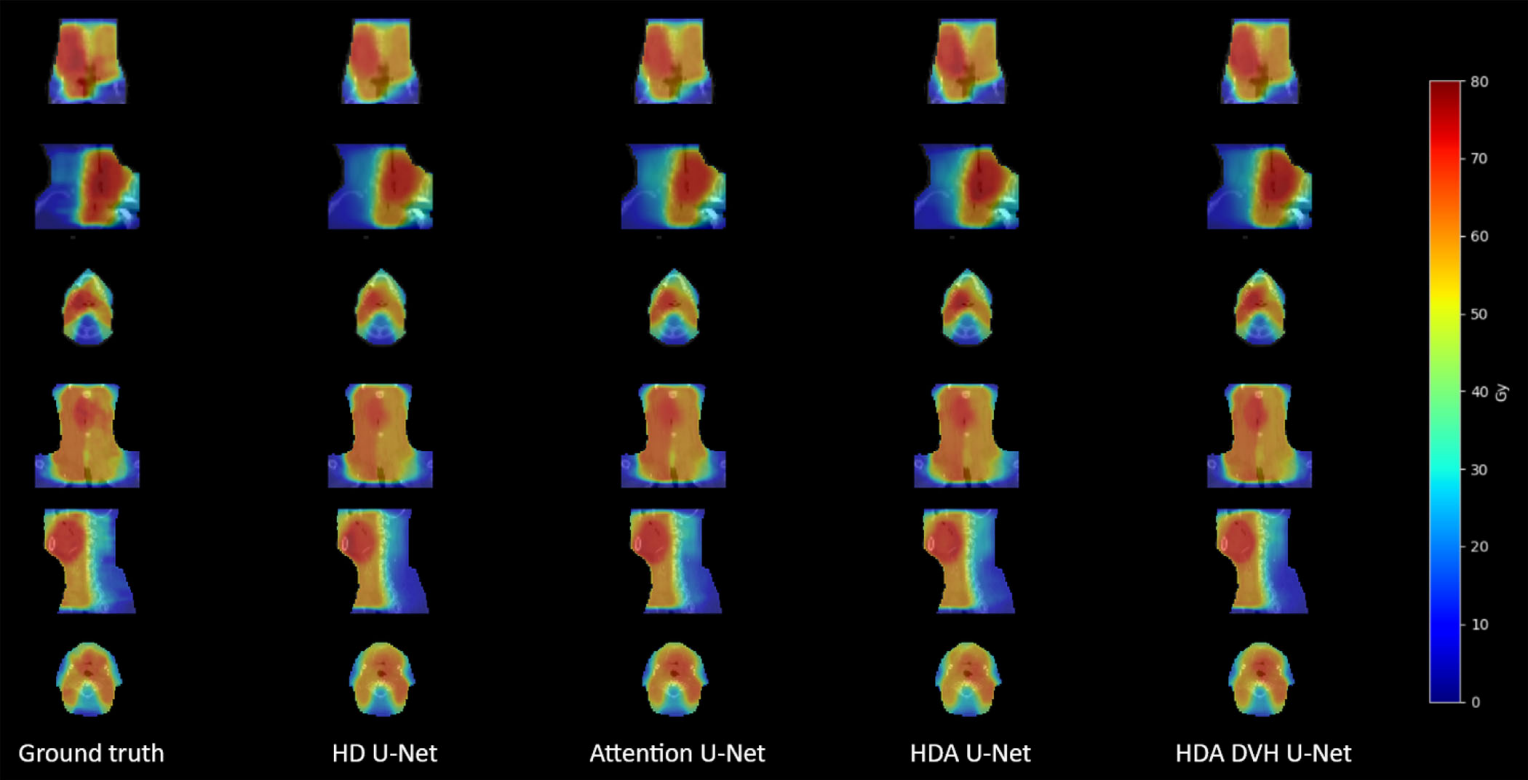
Dose examples overlapped with the input CT for two sample test patients of the OpenKBP dataset from ground truth, HD U‐net, attention U‐net, HDA U‐net, and proposed HDA DVH U‐net

Figure 7 shows dose prediction examples on sample patient cases from the public dataset. The dose distribution is close to ground truth across all architectures but tends to present higher levels of radiation around the throat for the first patient. On the same note, predictions are way smoother than the reference plans which demonstrate the inability of the MSE‐trained models to replicate perfect beam‐like trajectories. Overall, the proposed architecture yields the lowest average dose error in both examples with a plan within 4.0% and 2.2% average error of the reference distribution.

**FIGURE 8:**
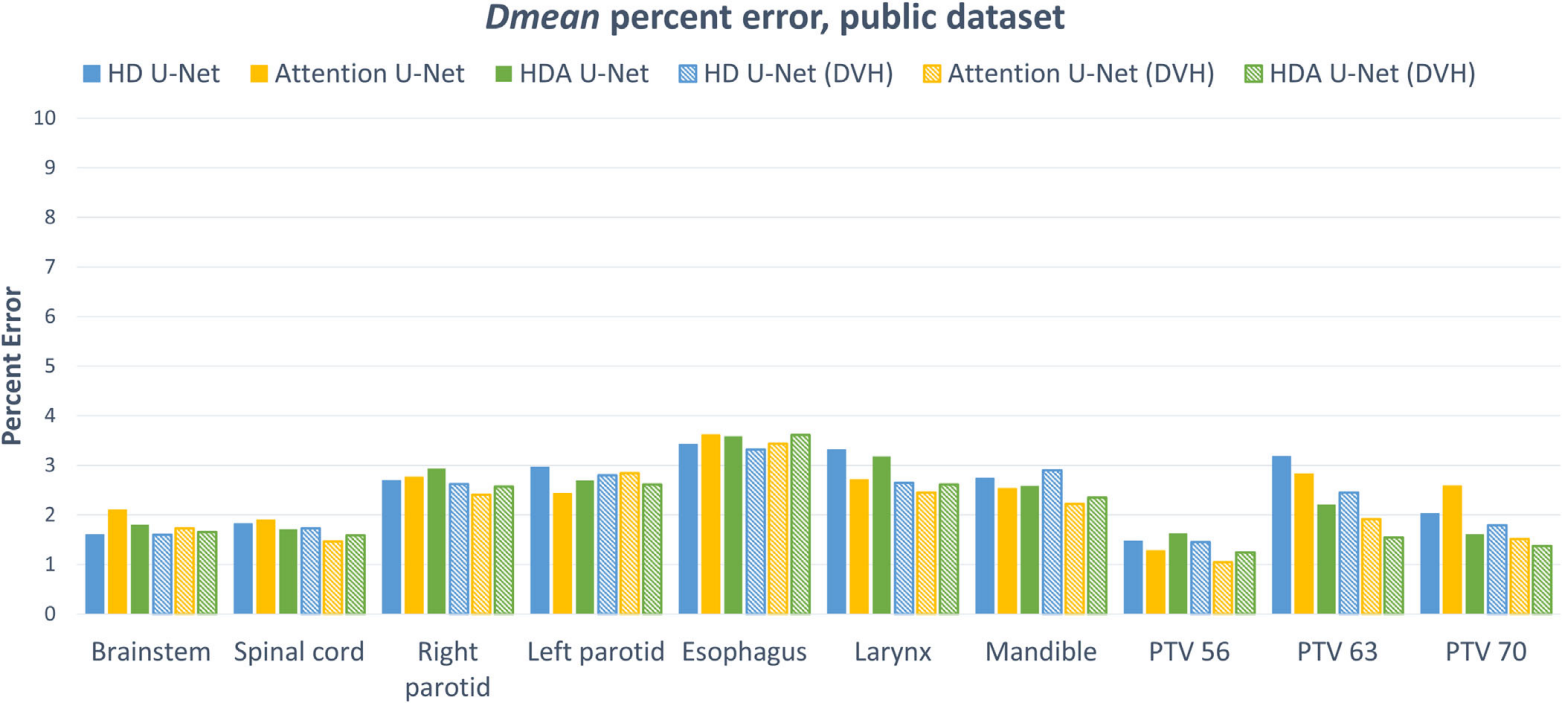
Dose mean percent error per structure for each model on the OpenKBP dataset

Figure 8 shows the average mean dose error for each architecture, on each structure of the public dataset. These are reported as a percentage of the prescribed dose. The error values are considerably lower for the public dataset, yet the same observations holds: the DVH loss positively impacts the dose error across all three architectures. The attention U‐net and the proposed method yield the lowest error on average on the OARs with a mean dose error of 2.4%, while the proposed method yields the lowest error on average on TVs with a mean dose error of 1.4% (1.5% for attention U‐net).

**FIGURE 9:**
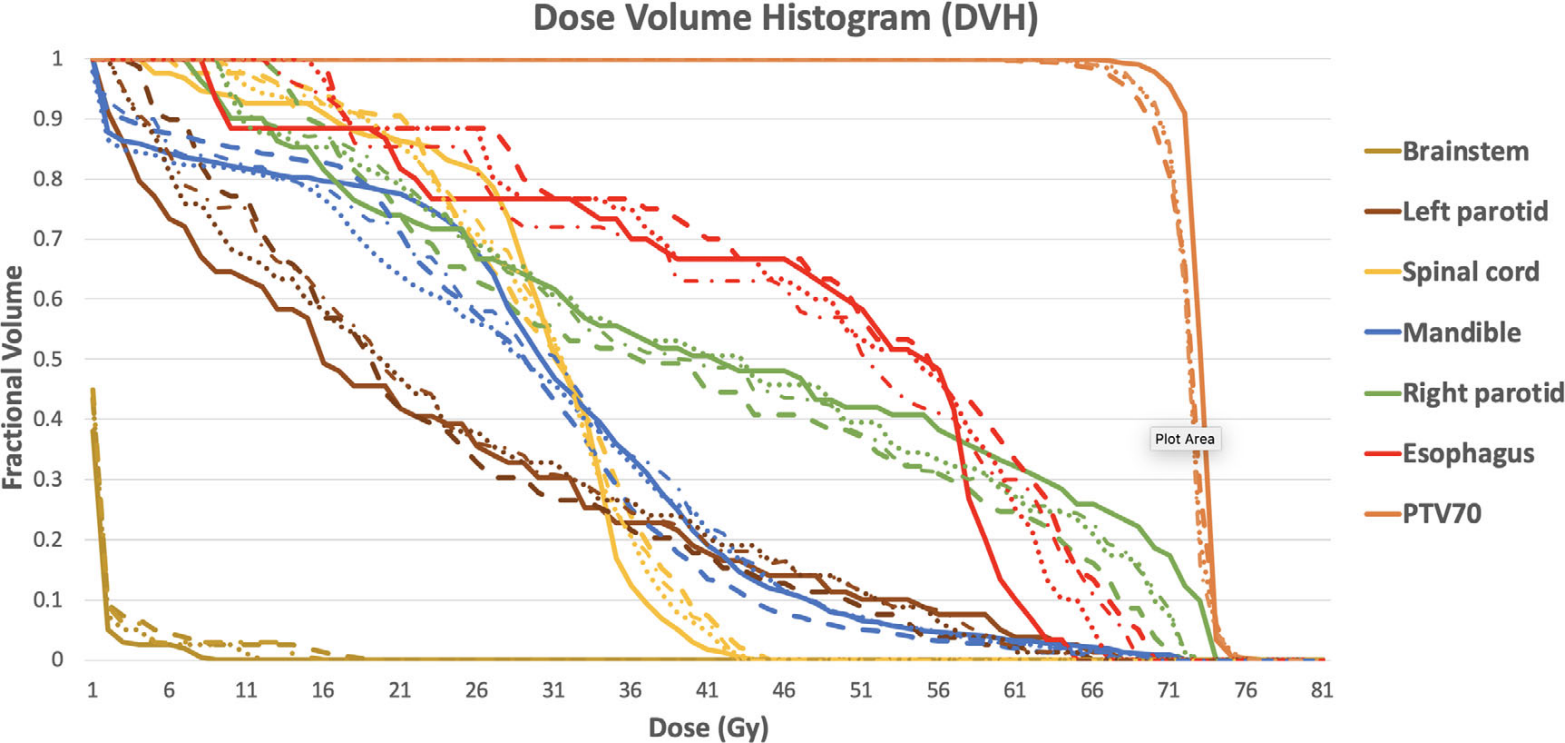
DVH curves for a sample patient of the OpenKBP dataset with doses from ground truth (solid line), HDA U‐net (dashed line), attention U‐net with DVH loss (dotted‐dashed line), and proposed HDA U‐net using DVH loss (dotted line)

Figure 9 shows sample DVH curves from a patient of the public dataset. For this sample patient, the proposed model's curves are consistently closer to the ground truth ones (except for the mandible between 15 and 30 Gy). Overall, the proposed method follows much closely the ground truth both in terms of value range and curve shape.

Finally, Table 6 shows a comparison of maximal and dose coverage values for the different OAR and PTVs for the entire datasets of H&N cancers cases, comparing manual dose plans with the proposed HDA U‐net with DVH loss.

**TABLE 6:** Comparison of dosimetry values of PTVs and OARs for patients with nasopharyngeal carcinoma in the manual plan and predicted plan from the HDA U‐net with DVH loss

Structure	Dose‐volume indices	Predicted plan	Manual plan	p‐values
PTV70	$D_{95}$ (Gy)	69.1 ± 0.01	68.6 ± 0.00	0.25
	H1	1.03 ± 0.01	1.07 ± 0.01	0.28
	CI	0.76 ± 0.02	0.80 ± 0.01	0.19
Brainstem	$D_{\max}$ (Gy)	59.7 ± 0.02	61.8 ± 0.05	0.31
Spinal cord	$D_{\max}$ (Gy)	41.6 ± 1.3	45.2 ± 0.8	0.17
Esophagus	$D_{\max}$ (Gy)	27.4 ± 2.3	29.8 ± 1.8	0.40
Mandible	$D_{\max}$ (Gy)	16.5 ± 2.7	18.0 ± 1.4	0.23
Left optic nerve	$D_{\max}$ (Gy)	45.3 ± 4.6	53.1 ± 3.7	< 0.01
Right optic nerve	$D_{\max}$ (Gy)	44.5 ± 5.8	50.9 ± 4.2	< 0.01
Left temporal lobe	$D_{\max}$ (Gy)	72.2 ± 3.3	70.0 ± 3.9	0.09
Right temporal lobe	$D_{\max}$ (Gy)	71.9 ± 3.5	70.6 ± 4.1	0.12
Left parotid	V30 (%)	56.3 ± 5.2	63.5 ± 6.4	< 0.01
Right parotid	V30 (%)	56.8 ± 4.9	64.0 ± 6.6	< 0.01
Oral cavity	V35 (%)	55.4 ± 7.9	56.1 ± 8.6	0.36
Larynx	V35 (%)	64.3 ± 6.4	63.4 ± 9.2	0.50

Abbreviations: DVH, dose‐volume histogram; HDA, hierarchically dense architecture; OARs, organs at risk; PTVs, planning target volume.

## 4 DISCUSSION

The models combining attention mechanisms with DVH loss demonstrated improved performance across most dosimetric measurements with a significant improvement over standard U‐net architecture in both Dmax and homogeneity scores. It is important to note that this is true on both datasets as well as with and without the DVH loss function. Furthermore, in most cases, the weighted MSE‐DVH improved the performance of the HD U‐net, the attention U‐net, and the proposed method. We believe this is an indication of the importance of using domain knowledge in deep learning when dealing with dosimetric predictions from medical imaging data. However, both the dense elements and the DVH objective significantly increase memory usage during training. The proposed HDA U‐net with the DVH loss is also a leaner model with significantly less parameters compared to the attention U‐net with DVH loss (3.4M vs. 26M parameters), leading to more efficient training and will tend to be more generalizable while generating clinically equivalent results. Every tested architecture is able to produce accurate dose plans within less than a second (depending on the size of the scans), which makes them very relevant for real‐time applications, such as adaptive radiotherapy procedures. As part of the clinical workflow, the developed method allows physicians to quickly access a feasible dose distribution and gives the dosimetrist a concrete objective. This should considerably decrease the number of iterations and thus shorten the overall treatment planning phase.

Adequately choosing what to provide the model as inputs plays an important part in developing a deep learning architecture for dose prediction. In recent dose prediction tasks, the patient CT is not always included as an input.^[^ ^11^ ^]^ In our experiments, however, its inclusion made a noticeable difference in terms of convergence. With the additional information included in the tomography, our networks were able to converge faster and more smoothly, achieving better performance. We ran ablation experiments in which the CT was omitted at prediction time, and the network would produce very diffuse plans as if it lacked boundaries. We believe the CT may offer additional points of reference for the network to accurately set radiation values and provide more defined gradients.

The public dataset allows a reproducible comparison to many other methods, whereas the in‐house dataset is more realistically variable and contains twice as many structures. We observe on the latter a wider score gap between the predicted dose and the reference dose. For example, the highest D95 coverage is within 3% of the reference score (0.95 vs. 0.98), while it is within 1% on the public data (0.98 vs. 0.99). The same is true for most metrics, especially the conformity measurements and underlines the impact of standardization on deep learning methods. One of the study goals is to evaluate the performance of the proposed model in various contexts. Systematically identifying and dealing with outliers remains an open problem in cancer treatment, meaning that outlier‐resistant architectures ought to be developed.

The addition of the DVH loss did improve performance overall; however, it seems to mostly benefit the dose delivered to the PTVs, especially for the in‐house dataset. The PTV channels may hold much needed information on the localization of the tumor and may drive the network to focus on them. On the other hand, nearby vital organs are also part of the dosimetric objective. That is why most knowledge‐based planning methods rely on constraints on both tumors and organs at risk to generate viable plans. Different ways of conveying this concept with deep learning methods include giving the model expected values for each structure^[^ ^29^ ^]^ or distance to target information.^[^ ^30^, ^31^ ^]^ In practice, instead of having structure masks containing binary values, the channels can hold these meaningful values for the given structure. The network can learn to pay attention to these values with the objective of improving the plan quality. In our experiments, both inclusions of expected values and distance‐to‐target information were effective with shallower 2D predictors. They were harder to fine‐tune for our deeper 3D architectures and often led to convergence and saturation issues. Another way to integrate these constraints in our deep learning pipeline would be to weight errors differently depending on the region they occur in. Liu et al. explored this idea through a modified MSE that gives higher weights to points that belong to smaller structures.^[^ ^32^ ^]^ As a future study, we plan to investigate this idea further by applying various weighting strategies to both the standard MSE and the DVH loss. We believe that placing more importance on specific organs might be particularly relevant for body areas that present a large variety of structures such as the H&N region.

This study had other limitations. Structures from the in‐house dataset were delineated by 13 different physicians. This has introduced a greater factor of variability within delineations and may lead to errors in the dose generation. Furthermore, the size of the datasets was fairly limited compared to other medical imaging tasks, which limits the generalization capabilities, as demonstrated by the varying levels of performance between the in‐house and public datasets.

The in‐house dataset was particularly challenging because it shows variations in prescribed doses, different fractions, different treatment objectives, artifacts, etc. It contains a realistic variety of cases, each delineated by one of 13 different physicians. This represents a variety of annotation styles, all of which may be equally correct, that the model must learn to generalize from when producing a dose prediction. Similarly, there may not be a single “ground truth” for dose prediction as different physicians may differ in their planning approaches. For a model to produce useful outputs for a physician, it must be capable of producing outputs that match that physician's style with specific case‐by‐case considerations and thus require minimal adjustment. In future work, we will explore the possibility to modify outputs to match the physician's style. Balagopal et al. explored this idea by collecting annotations from different physicians and training a model with a separate decoder for each physician.^[^ ^33^ ^]^ Further customization of the output can be achieved by developing a semiautomated method for the physician to submit corrections of the output to the model in an iterative fashion. One could even allow the physician to set constraints that are provided to the model, as in hypernetworks that take hyperparameters as input.^[^ ^34^ ^]^ These constraints could be per‐region target dose values or even target dose values for specific pixels.

## 5 CONCLUSION

We developed a hierarchically dense attention U‐net architecture, HDA U‐net, to predict 3D dose distributions for H&N cancer patients. Our architecture integrates dense elements and soft attention gates in a 3D U‐net architecture. We explored the addition of a clinical objective with a weighted MSE‐DVH loss function and proved it could be relevant in H&N cancers. We compared our proposed model with two variants of the U‐net: the HD U‐net and the attention U‐net. Both methods combining attention mechanisms and DVH loss yielded the best results on two distinct datasets, showing improved generalization capabilities. Using the DVH objective, the HDA U‐net outperformed the HD U‐net model on all metrics and outperformed the attention U‐net on homogeneity while maintaining high conformity levels and a significantly lower maximum dose. The dose distributions can be generated within 1 s, making it very applicable for near real‐time purposes and allowing physicians to quickly produce a feasible goal for the dosimetrist to use as reference for planning. In future work, we plan to explore different weighting strategies to further improve the quality of the plans around specific organs and include the ability to match a physician's style to better cope with variability within delineations.

## CONFLICT OF INTEREST

The authors have declared no conflict of interest.

## AUTHOR CONTRIBUTION

Samuel Cros participated in data curation, conceptualization, methodology, formal analysis, visualization, writing, and editing. Hugo Bouttier participated in data curation, methodology, software, and visualization. Phuc Felix Nguyen‐Tan participated in formal analysis and project administration. Eugene Vorontsov participated in methodology, software, and visualization. Samuel Kadoury participated in formal analysis, writing and editing, resources, project administration, and funding acquisition.
